# Image-based response assessment of liver metastases following stereotactic body radiotherapy with respiratory tracking

**DOI:** 10.1186/1748-717X-8-24

**Published:** 2013-01-30

**Authors:** Hajer Jarraya, Xavier Mirabel, Sophie Taieb, Sylvain Dewas, Emmanuelle Tresch, Francois Bonodeau, Antoine Adenis, Andrew Kramar, Eric Lartigau, Luc Ceugnart

**Affiliations:** 1 Department of Radiology, CLCC Oscar Lambret, 3 rue Frédéric Combemale, BP 307, Lille cedex, 59020, France; 2 Department of Radiotherapy, CLCC Oscar Lambret, 3 rue Frédéric Combemale, BP 307, Lille cedex, 59020, France; 3 Department of Methodology and Biostatistics, CLCC Oscar Lambret, 3 rue Frédéric Combemale, BP 307, Lille cedex, 59020, France; 4 Department of digestive oncology, CLCC Oscar Lambret, 3 rue Frédéric Combemale, BP 307, Lille cedex, 59020, France

**Keywords:** Stereotactic body radiotherapy, Cyberknife, Imaging, Liver metastasis, Response assessment

## Abstract

**Objective:**

To describe post-CyberKnife® imaging characteristics of liver metastases as an aid in assessing response to treatment, and a novel set of combined criteria (CC) as an alternative to response according to change in size (RECIST).

**Subjects and Methods:**

Imaging data and medical records of 28 patients with 40 liver metastases treated with stereotactic body radiotherapy (SBRT) were reviewed. Tumor size, CT attenuation coefficient, and contrast enhancement of lesions were evaluated up to 2 years post SBRT. Rates of local control, progression-free survival, time to progression, and overall survival according to RECIST and CC were estimated.

**Results:**

Complete response (CR) was 3.6% (95% CI: 0.1–18%) and 18% (95% CI: 6–37%) according to RECIST and combined criteria, respectively. Two progressive diseases and two partial responses according to RECIST were classified as CR by the combined criteria and one stable response according to RECIST was classified as progressive by CC (Stuart-Maxwell test, p = 0.012). The disease control rate was 60.7% (95% CI: 41–78%) by RECIST and 64% (95% CI: 44%–81%) by CC.

**Conclusion:**

Use of response criteria based on change in size alone in the interpretation of liver response to SBRT may be inadequate. We propose a simple algorithm with a combination of criteria to better assess tumor response. Further studies are needed to confirm their validity.

## Introduction

Stereotactic body radiotherapy (SBRT) is a technique that allows the delivery of a precise dose to a tumor while sparing adjacent normal tissues. Its use for cerebral metastases has shown high local control rates of more than 80–90% [1]. The use of stereotaxy in the treatment of intra-abdominal organs, however, has been hampered by the movement of these organs along with respiration. Robotic SBRT with the CyberKnife® System (Accuray Incorporated, Sunnyvale, CA) is a technique that allows tracking of the respiratory motion, thus enabling delivery of the dose with accuracy even while the patient breathes freely [2].

The standard first-line treatment for liver metastases is surgery. Yet less than 20% of liver metastases are surgically removable because of their often too large size. The difficulty of the surgical access can be due to anatomic localization of the tumor or other associated comorbidities present with the patient [3]. Other available therapeutic options for inoperable liver metastases are intra-arterial chemo-embolization, Y90 radio-embolization, radiofrequency ablation, and ethanol injection [3,4]. Stereotactic body radiotherapy (SBRT) is an additional alternative modality that has emerged thanks to recent advances in medical technology and robotics, and appears to result in favorable liver tolerance and good response rates [5-9].

Following the treatment, monitoring the response to the treatment with imaging is of utmost importance, because under- or over-interpretation of the response may have severe consequences, such as subjecting the patient to unnecessary chemotherapy, or an unnoticed recurrence. Hepatic tumors display certain unusual characteristics upon treatment with SBRT, therefore, interpretation of response using the usual size-based guidelines has been difficult in the case of liver treatments.

In this article, we describe post-therapeutic transformations of secondary hepatic lesions treated with CyberKnife with the objective of identifying new criteria of early response and progression by incorporating these morphological changes.

## Subjects and methods

### Patients and data

Between July 2007 and November 2010, 103 patients with liver metastases, ineligible for surgery or radiofrequency ablation, underwent SBRT at our center. Inclusion criteria were: WHO performance status score less than 3, four hepatic lesions or less, and initial lesion size smaller than 100 mm. The delivered dose was 40 Gy in four fractions at the beginning, and then increased to 45 Gy in three fractions.

Among these patients, 28 patients were eligible for this study: initial and at least two successive follow-up contrast-enhanced CT (CECT) examinations after treatment, no concomitant chemotherapy, a minimum initial target lesion size of 10 mm, as for RECIST criteria.

The medical records and image data were reviewed retrospectively by our internal review board including one radiologist and two radiation oncologists.

Portal phase images for 28 patients who presented with 40 initial lesions and 163 follow-up evaluations on CECT scans (performed every 3 months after treatment) were reviewed. CT scans were obtained with a 16-detector row CT scanner (Sensation 16; Siemens Medical Solutions). Detector collimation was 1.5 mm. Tube potential was 120 kV, tube current-time product was 185 mAs, pitch was 0.75, section thickness was 5 mm, and reconstruction increment was 5 mm.

### Image analysis

#### Lesion size

Lesion sizes were measured at the longest cross-sectional dimension of each lesion at each time point. Response for each lesion (LR) was evaluated at each of the 163 follow-up evaluations for each of the 40 lesions as the percent change from the pretreatment evaluation and classified according to the same cut-off points as used for RECIST [10]. The RECIST criteria was also calculated for each patient ignoring other metastatic sites: complete response (CR), disappearance of all hepatic lesions; partial response (PR), more than a 30% decrease in the sum of the longest diameter of hepatic lesions from baseline; stable disease (SD), neither partial response nor progressive disease; and progressive disease (PD), more than a 20% increase in the sum of the longest diameter of hepatic lesions from nadir [10].

#### CT Attenuation coefficients

CT attenuation coefficient (density) of each lesion was measured in Hounsfield units (H) by drawing a region of interest circumscribing the margin of each lesion (the hypodense area). In patients scanned using the triphasic technique, the portal venous phase images were used for the lesion density measurements. Before measuring the lesions’ CT attenuation coefficients, the reproducibility of different monitors (CT operator’s console [Siemens] and workstation in a radiologist’s office) were tested using acrylic, water, and air phantoms. No significant differences were found in the CT attenuation coefficients of the phantoms measured on different monitors.

Necrosis was defined as non-enhancing tissue. A maximum increase of 10 H on CT after contrast administration (comparing non-enhancing and enhancing CT scans at the same level of the lesion) was considered necrotic tissue, since this was considered insignificant [11]. Contrast enhancement was categorized based on its shape as explained in the results section under imaging findings.

### Treatment response

Two methods were used to assess treatment response of each lesion: the size-based criteria, which was based solely on change in size, and a combination criteria based on the enhancement pattern of the lesion as well as its size. At each follow-up evalaution, a first analysis concerning each individual lesion and a second analysis concerning the status of all lesions for each of the 28 patients (RECIST) were performed. The combination criteria (Table 1) consisted of the following: complete response, disappearance of all lesions or total necrosis (independent of size) (cCR); partial response, more than a 30% decrease in lesion size and no total necrosis and no lobulated enhancement (cPR); stable disease, neither cPR nor cPD (cSD); and progressive disease, more than a 20% increase in lesion size and no total necrosis, or the presence of lobulated enhancement (cPD). For patients with multiple lesions, RECIST criteria was used for all lesions and patients were classified as progressive if a lobulated enhancement was seen for at least one lesion. These patients were classified in complete response only if all of the lesions were necrotic. 

**Table 1:** Treatment response according to lesion size, contrast enhancement, necrosis, and combined criteria

**Treatment response**	**Lesion size**	**Contrast Enhancement**	**Necrosis**	**Combined criteria**
Complete response	Disappearance of lesion	-	Total necrosis	Disappearance of lesion **OR** Total necrosis
Partial response	≥ 30% decrease	-	-	≥ 30% decrease
Stable disease	< 30% decrease or <20% increase	-	-	Neither complete, nor partial nor progressive lesion
Progressive disease	≥ 20% increase	Lobulated thick enhancement	-	≥ 20% increase **OR** Lobulated thick enhancement

### Statistical analysis

Demographics and disease characteristics are presented with frequencies and percentages for categorical variables. Continuous variables are presented with medians and ranges.

Response rates are presented with a 95% confidence interval calculated from the binomial distribution. The two classification systems (change in lesion size and combined criteria) were compared using the Stuart-Maxwell test at each of the 163 follow-up evalaution as well as for the 28 patients.

All time-to-event analyses were calculated from the start of treatment. Overall survival times were calculated until death or the cut-off date (November 30, 2010), whichever came first. Time to progression for each patient was calculated until the criteria for progression were met for both the RECIST and combined criteria. Non-progressive patients and those deceased from unrelated causes were censored at last follow-up or at date of death. Progression-free survival was calculated until the criteria for progression were first met or until death. Patients who did not progress or did not die were censored at last follow-up. Survival rates were estimated with the Kaplan-Meier method.

## Results

Twenty-eight patients presented with 40 lesions. Median age was 63.5 years (range, 23–81 years) and 64% were men (Table 2). The primary was colorectal cancer in 19 patients (68%), breast cancer in one patient (3.5%), and other sites for eight patients (28.5%): pneumoblastoma, melanoma, leiomyosarcoma, bronchial carcinoma, pulmonary epidermoid carcinoma, epidermoid carcinoma of the cavum, and endocrine carcinoma of the pancreas. The number of lesions by patient were as follows: 19 patients (68%) had one lesion, seven patients (25%) had two lesions, one patient (3.5%) had three lesions, and one patient (3.5%) had four lesions. The median number of evaluations was 6 (range 3 – 28). 

**Table 2:** Demographic and disease characteristics

**Demographic and disease characteristics**	
**Age**, median (range)	63.5 (23–81)
**Sex** (n, %)	
M	18 (64.3%)
F	10 (35.7%)
**Localization** (n, %)	
Colorectal	19 (68%)
Breast	1 (3.5%)
Other	8 (28.5%)
**Number of lesions** (n, %)	
1	19 (68%)
2	7 (25%)
3	1 (3.5%)
4	1 (3.5%)

### Imaging findings

#### Contrast enhancement

**Figure 1:**
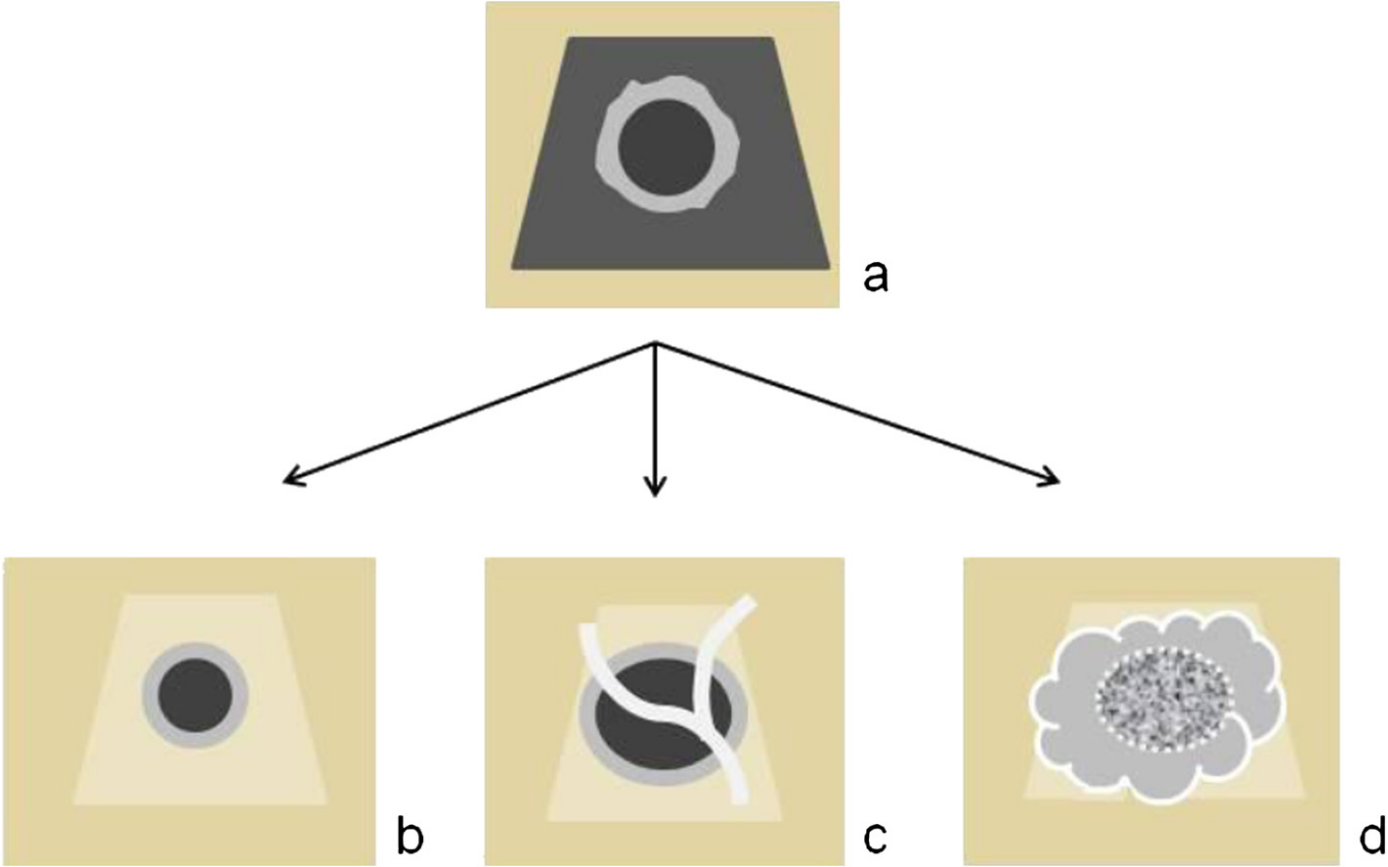
Schematic representation of the target-shaped lesion at 3 months (a), note the hypodense area and the surrounding rim enhancement; after 3 months in case of local control(b,c), the lesion becomes smaller(b), retains its size, or even grows surrounded by a thin rim enhancement with total necrosis (c); and (d) in case of local recurrence, note the heterogeneous center surrounded by lobulated irregular thick enhancement; Note the shrinkage of the hypodense area that becomes hyperdense in all cases.

At first follow-up, the shape of the lesion was almost always the same: a hypodense center surrounded by a peripheral rim enhancement localized in a large hypodense area with a clearly defined border between the irradiated parenchyma and the normal liver, as schematized in Figure 1a. This large hypodense area was seen in 31/40 lesions. This depended mostly on the interval between the treatment and the first CT; it was no longer observable after 2 months. In one case of hepatic steatosis, this area was observed displaying hyperdensity. On further follow-up, it became increasingly isodense with the normal hepatic parenchyma, and then became hyperdense again while continuously shrinking. This enhancement pattern was present in all patients treated with CyberKnife regardless of prognosis. 

**Figure 2:**
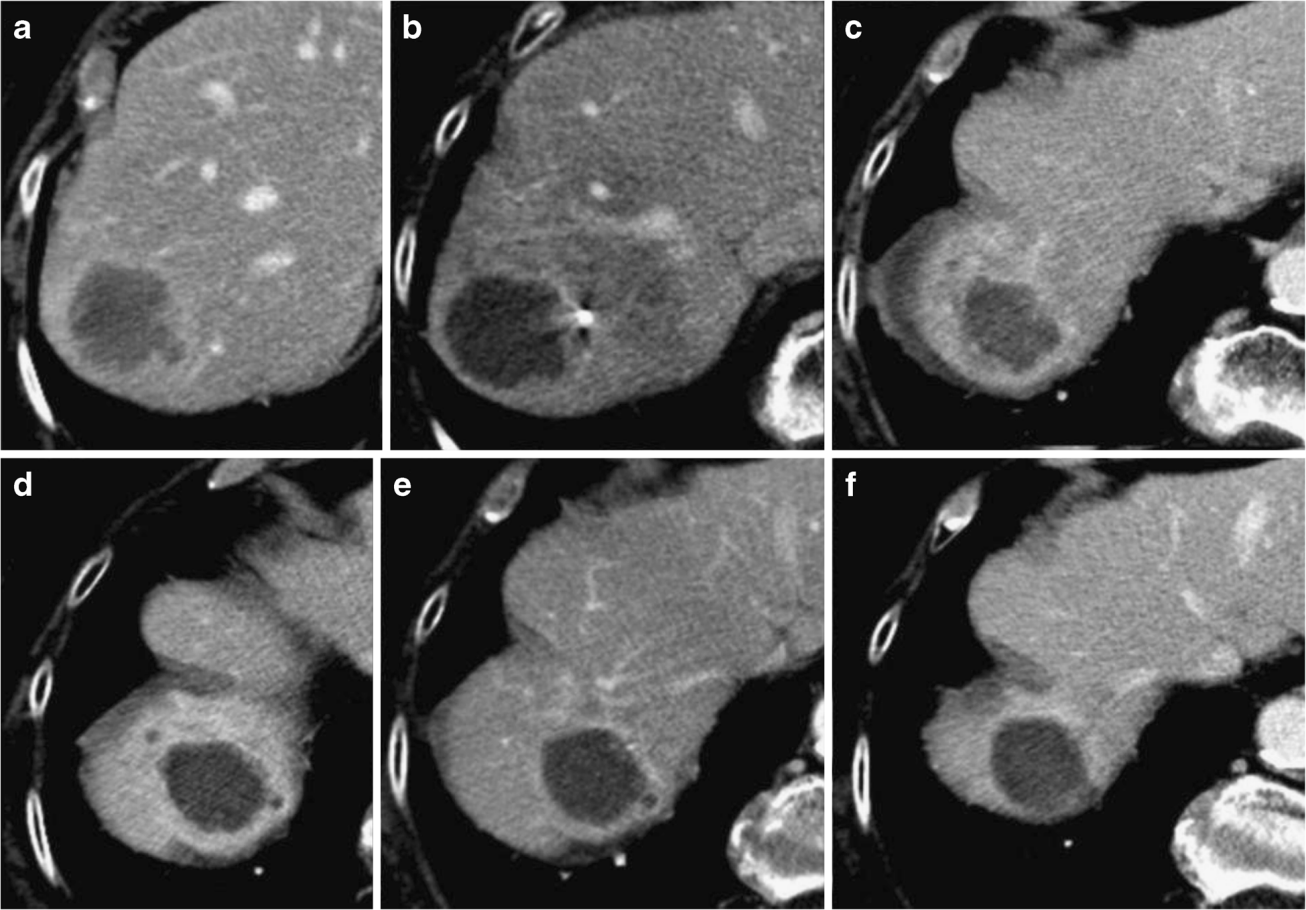
**Complete response after SBRT for a liver metastasis over successive follow-ups. **Before treatment (**a**); 2 months later (**b**), we observe changes of the initial phase: target-shaped lesion surrounded by a peripheral enhancement and located in a hypodense area with well delineated demarcation between irradiated and normal liver; CT slices performed at portal phase at 5 months (**c**); 9 months (**d**); 12 months (**e**); and 24 months later (**f**) show that the center is better circumscribed by a thin rim enhancement, the hypodense area progressively shrank and became hyperdense.

**Figure 3:**
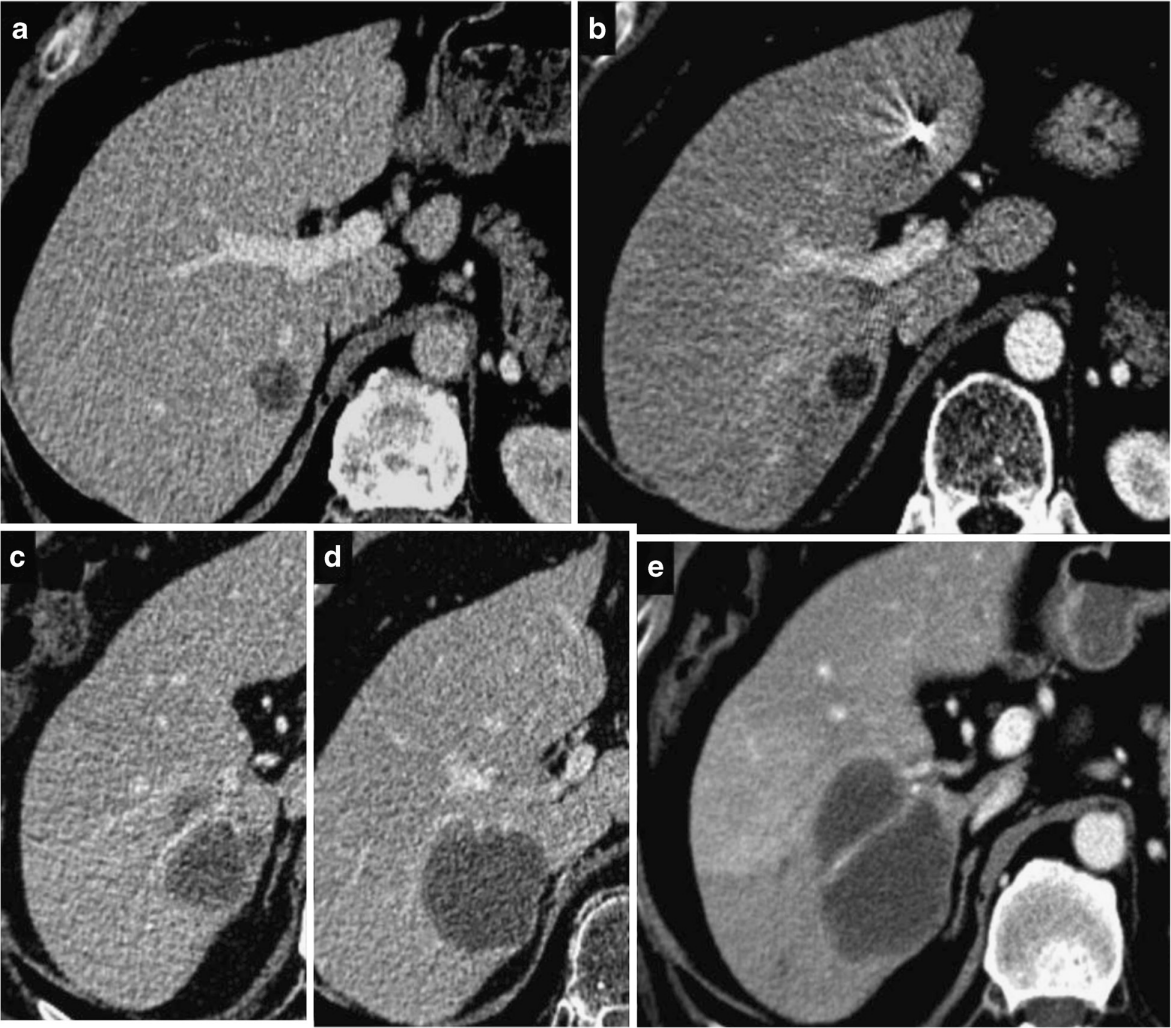
**Pseudo- progressive lesion after SBRT, before treatment (a), 2 months later (b), 6 months later (c, d), and 9 months later (e). **CT slices show the progressive size increase with a completely necrotic lesion.

**Figure 4:**
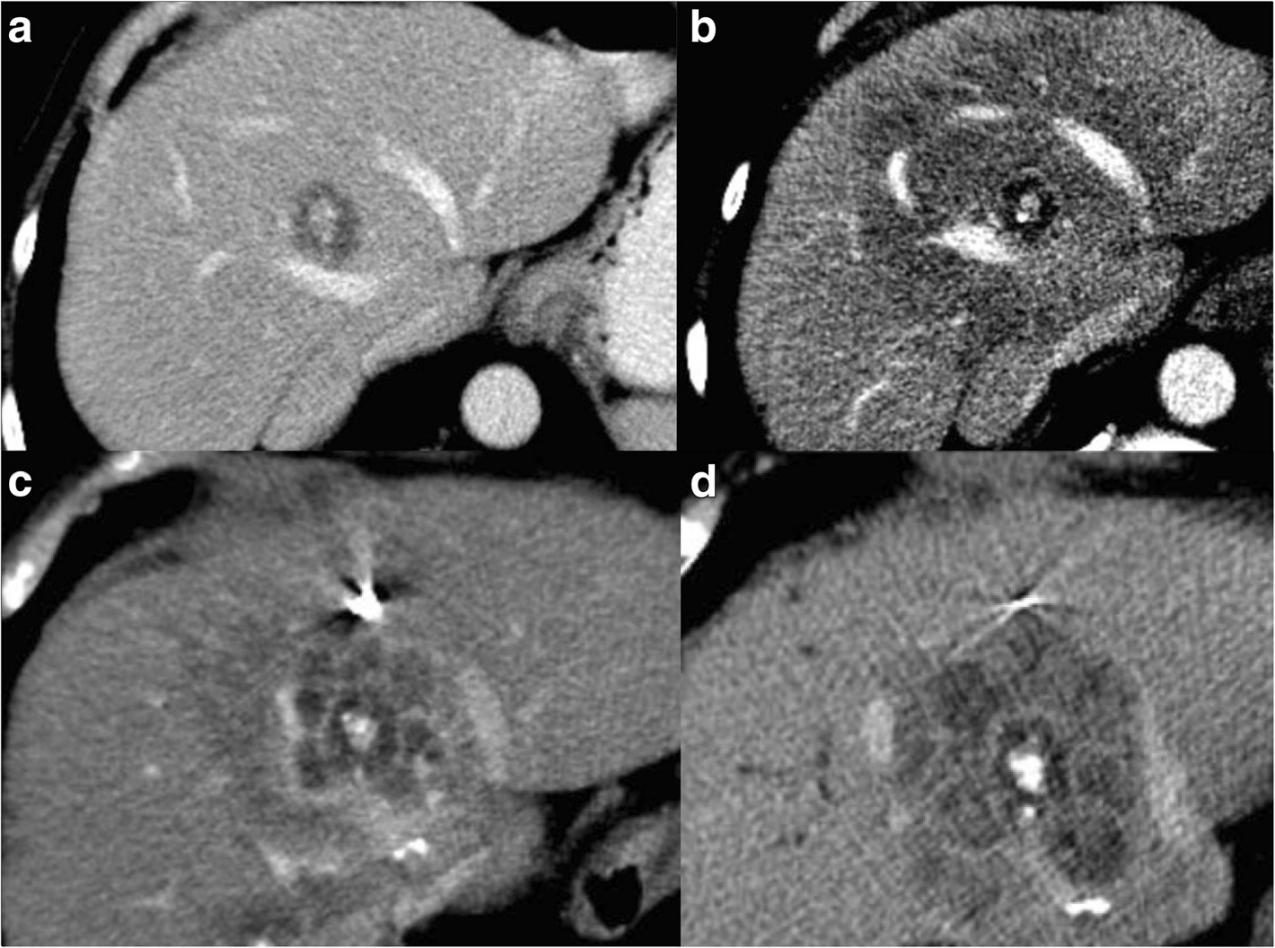
Progressive lesion after SBRT; a liver metastasis with a calcified center before treatment (a), 2 months later (b) we observe the changes of the initial phase as described, 4 months (c) and 6 months later (d) CT slices show the Daisy Sign: a lobulated thick peripheral enhancement.

After treatment, every lesion displayed a thin rim enhancement at least in one of successive follow-up images (Figures 2 and 3). On long-term observation, this appearance remained stable in 30 lesions or, otherwise, assumed a lobulated and thick manifestation resembling a daisy in 10 lesions (Figure 4). Lobulated thick enhancement was first seen at 3.7 months (median 7.6 months, mean 9 months) after treatment. This enhancement increased with time in every lesion that had it. 

### Treatment response by the lesion

Change in tumor size at each of the 163 follow-up evaluations along with the necrosis and contrast enhancement information are presented in Table 3. Size-based criteria identified 11 CR, 71 PR, 52 SD, and 29 PD. According to the combined criteria, there were 23 cCR, 67 cPR, 45 cSD, and 28 cPD. Among the 71 lesions categorized as PR (decrease of more than 30% in size), four lesions were necrotic, 45 were cSD, and 11 were cCR as judged by the combined criteria. All cPR and cSD lesions showed a target-shaped morphology and were distinctly surrounded by a well pronounced rim enhancement, indicating local control, as shown in Figures 1b and 2.

**Table 3:** Lesions response according to change in tumor size and combined criteria

**Lesion response (N=163)**	**Change in tumor size**				
	**CR**	**PR**	**SD**	**PD**	**Total, n (%)**
**Combined criteria**					
cCR	11	4	5	3	23 (14.1%)
cPR	0	67	0	0	67 (41.1%)
cSD	0	0	45	0	45 (27.6%)
cPD	0	0	2	26	28 (17.2%)
Total, n (%)	11 (67.5%)	71 (43.6%)	52 (31.9%)	29 (17.8%)	

A total of eight (5%) lesions were classified as *pseudo-progressive***,** either because they stabilized after a period of growth or were completely necrotic despite their growth in size (Figures 1c and 3). These lesions were among those classified as progressive (three lesions) or stable (five lesions) according to the size-based criteria. In two cases, blood vessels were observed to penetrate these lesions without deformation, attesting to their necrotic nature.

According to the combined criteria, there were 28 (17%) *progressive lesions***,** 26 of which were diagnosed based on a persistent size increase observed over successive imaging and two lesions, although stable in size, showed a lobulated thick heterogeneous enhancement. All of the progressive lesions eventually exhibited a lobulated thick heterogeneous enhancement, indicating strong likelihood for failure (Figures 1d and 4).

### Treatment response by patient

**Figure 5:**
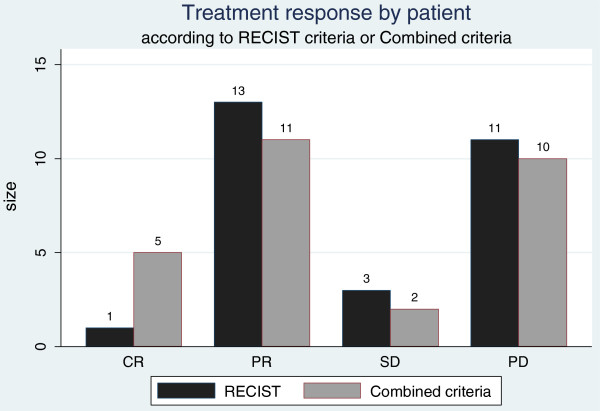
Treatment response by patient according to RECIST and Combined criteria.

The complete response rate was 3.6% (95% CI: 0.1–18%) according to RECIST criteria and 18% (95% CI: 6–37%) according to the combined criteria. The distribution of CR between the two sets of criteria were significantly different (McNemar test, p = 0.046): two PDs and two PRs according to RECIST were identified as CR according to the combined criteria (Table 4; Figure 5). The objective response rate was 50% (95% CI: 31–69%) according to RECIST criteria and 57% (95% CI: 37–76%) according to the combined criteria. The disease control rate was 60.7% (95% CI: 41–78%) according to RECIST and 64% (95% CI: 44–81%) according to the combined criteria. The classification of progressive disease by the two categories was not significantly different. 

**Table 4:** Treatment response using RECIST or combined criteria

**Response rates by patient (N=28)**	**RECIST criteria**				
	**CR**	**PR**	**SD**	**PD**	**Total, n (%)**
**Combined criteria**					
cCR	1	2	0	2	5 (17.9%)
cPR	0	11	0	0	11 (39.3%)
cSD	0	0	2	0	2 (7.1%)
cPD	0	0	1	9	10 (35.7%)
Total, n (%)	1 (3.6%)	13 (46.4%)	3 (10.7%)	11 (39.3%)	

CR: complete response; PR: partial response; SD: stable disease; PD: progressive disease.

#### Overall survival

Median follow-up was 23.1 months (range 13 – 33). Eight patients died (28.6%), six from disease progression and two from other causes (one respiratory failure, one hepatic failure leading to encephalopathy). Median overall survival was not reached. The 1- and 2-year survival rates were 96.4% and 67.8%, respectively (Table 5). 

**Table 5:** Survival endpoints using RECIST or combined criteria

**Survival endpoints**	** RECIST**	** Combined criteria**
Overall Survival		
Death	8	
Median [CI 95%]	-	
OS rate, % [CI 95%]		
12 months	96 [77.2-99.5]	
18 months	72. [51–86]	
24 months	67 [44–82]	
Progression-Free Survival		
Progression or Death (every cause)	14	15
Median [CI 95%]	19.3 [8.0-…]	19.1 [12.3-…]
PFS rate, % [CI 95%]		
12 months	68 [47–82]	71 [51–85]
18 months	56 [36–72]	52 [32–69]
24 months	47 [27–65]	43 [24–61]
Time to Progression		
Progression or death from disease progression	13	13
Median [CI 95%]	-	19.3 [12.3-…]
TTP rate, % [CI 95%]		
12 months	68 [47–82]	71 [51–85]
18 months	60 [40–76]	60 [39–75]
24 months	51 [30–68]	49 [28–67]

#### Progression-free survival

**Figure 6:**
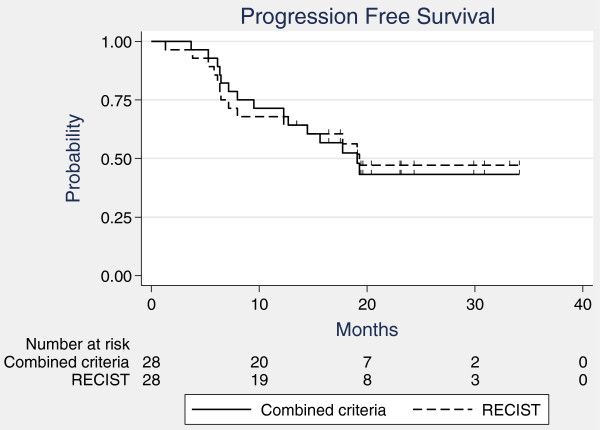
Progression-free survival according to combined criteria and RECIST criteria.

Median progression-free survival for the 28 patients was 19.3 months according to RECIST and 19.1 months according to combined criteria (Figure 6). The 1- and 2-year progression-free survival rates were 67.9% and 47.2% using RECIST, and 71.4% and 43.2% using combined criteria, respectively. 

#### Time to progression

Thirteen patients had progressive disease or died from disease progression according to both classifications. Median time to progression was not reached according to RECIST and was 19.3 month according to combined criteria. The 1- and 2-year time-to-progression rates were, respectively, 67.9% and 50.8% using RECIST, and 71.4% and 49.4% using combined criteria.

## Discussion

Assessment of response to an oncologic treatment is usually based on international guidelines such as the World Health Organization (WHO) guidelines or the Response Evaluation Criteria in Solid Tumors (RECIST) group [10,12]. These criteria are based on the assessment of the change in tumor size after treatment. However, they appear to be less adapted in assessing the efficacy of targeted therapy, such as those for gastrointestinal stromal tumors treated with anti-angiogenic agents [13].

Functional imaging such as FDG Positron Emission Tomography (PET), contrast enhancement ultrasound (CE US), and diffusion-weighted Magnetic Resonance Imaging (MRI) are sensitive methods which allow an early assessment of metabolic response for different treatments [14-17]. However, these methods are available at only a limited number of institutions.

Along with other applications, SBRT is an excellent noninvasive option for the treatment of inoperable hepatic tumors [4,7].

Imaging studies play an integral role in monitoring response to SBRT [18,19]. Traditionally, a decrease in tumor size according to the RECIST criteria has been considered CT evidence of treatment response 9, 10]. These criteria are the international standard to assess response. However, they are often inadequate in interpreting the results following local or targeted therapy [13,20-24].

Acknowledging the limitations of the size criteria, some experts proposed considering lesion enhancement in the evaluation of therapeutic response of malignant tumors, such as modified RECIST (mRECIST) for hepatocellular carcinoma (HCC) [24], the CHOI criteria for gastrointestinal stromal tumors (GIST) [13], or the CHUN criteria for colorectal metastases treated with bevacizumab [25].

In this study, we observed the profile evolution of 40 liver lesions following SBRT. This way, we were able to objectively distinguish the responsive lesions from the progressive ones. The responsive lesions included those with complete necrosis, complete disappearance, partial response, or stabilization; and also the pseudo-progressive lesions that initially increased their size or remained stable and yet were completely necrotic.

Our method for response evaluation combines size, enhancement, and necrosis criteria based on the retrospective observation of SBRT-treated hepatic lesions. Lesions were classified into four categories: CR, PR, and SD for local control, and PD for local recurrence. A response was considered complete if a lesion disappeared, or the remaining tissue appeared completely necrotic regardless of its size. Also according to combined criteria, the occurrence of a lobulated enhancement pattern around the lesion was considered PD regardless of any change in lesion size. A size increase according to RECIST without total necrosis was also considered as progressive disease. For the combined criteria, partial response and stable disease are identical to their counterparts in the RECIST criteria. Owing to measurements unreliability of CT attenuation coefficient for small lesions, we did not consider necrosis criteria for lesions ≤15 mm [26].

During the course of the study, we observed that if the RECIST criteria were to be applied without question, this may result in misinterpretation of some of the responses to the treatment. For instance, a progressive lesion according to the combined criteria was deemed stable according to RECIST. Some of the perfectly responsive lesions increased in size and were classified as progressive according to RECIST, but we were able to attribute this increase to necrosis, cystic degeneration, hemorrhage, or edema [27]. Therefore, size increase was not enough in and of itself as a criterion to indicate progression. In our study, two patients had lesions which became completely necrotic but increased in size and would have been erroneously categorized as PD based on the traditional size criteria. These lesions did not show any enhancement unlike the really progressive lesions that increased in size yet stayed solid. Therefore, it is sometimes necessary to use more diverse criteria based on the morphological characteristics of a lesion that can be observed on CT.

The lobular enhancement pattern we defined in the results section is a morphologic sign based on the lesion enhancement shape that is easy to identify by contouring the outline of a lesion. This pattern seemed to be a sensitive and specific sign for assessment of local relapse, which well correlated with size changes in 9/10 patients. Lobulated enhancement could sometimes allow earlier identification of local recurrence. In 2/10 patients, the lobulated enhancement appeared earlier than size progression, at the same time for 7/10 patients, and later in 1/10 patient. In this last case, the telltale sign was difficult to spot because of the lesion’s location that contained no surrounding parenchyma. The determination of the sensitivity and specificity of this pattern needs further studies with a larger number of progressive lesions. This pattern may have been detected earlier than the enlargement in size if an intermediate CT scan between 6th and 9th months had been recorded. In fact, on analysis of the progression-free survival curves, the drop in the curve with the two methods of evaluation occurring between the 6th and 9th months suggests that this period may be important in the surveillance of the lesions, and the insertion of an extra CT scan during this period may be instrumental in earlier detection of a possible failure. This way, an earlier medical intervention could also be initiated.

Total necrosis was also easy to spot by measuring with an objective method the density of the lesion before and after contrast injection: a difference ≤10 H indicated no enhancement, therefore, necrotic characteristics. This method could not be used for small lesions that were <15 mm, in which case the measurements became unreliable. The necrosis criterion allowed the identification of complete response cases that were erroneously classified as progressive based on their size increase.

The thin rim enhancement was seen in all lesions in our study. This finding is likely due to the presence of granulation tissue related to inflammatory response to the treatment [28].Our findings are consistent with the literature. For instance, Olsen *et al.*[28] have described a zonal pattern of SBRT injury in two patients treated by SBRT and who underwent surgery. Herfarth *et al.*[29] have described three different types of reaction based on the time of imaging after SBRT corresponding to the histological changes seen in veno-occlusive disease (VOD). The results of our study matches those of Herfarth fairly well. The VOD that was initially seen as a hypodense area would become fibrotic, becoming smaller and denser as seen on successive follow-ups. In only one patient who had a case of fatty infiltration of the liver, the CT showed a relatively increased density of the surrounding area in the portal phase. This has also been described by Yamasaki in 2/31 patients treated by conformal radiation therapy [30]. A distinct border between the target and normal parenchyma was always visible at the treatment margin. This phenomenon also attested to the CyberKnife beams’ accuracy of distribution.

There was no statistical difference in classification of progressive disease using RECIST criteria and combined criteria due to the small number of progressive lesions (n = 10 for the combined criteria and n = 11 for RECIST). Nevertheless, the use of the combined criteria led to a more accurate detection of response and progression than the use of RECIST criteria. Among patients with RECIST-identified progressive lesions (n = 11), necrosis and enhancement criteria allowed to differentiate between progressive (n = 9 PD) and pseudo-progressive lesions (n = 2 CR). A display of complete necrosis proves a lesion is in complete response. On the other hand, the lobulated thick enhancement can be useful for confirmation of progression when progression is suspected based on the size criterion.

Although the response rates excluding SD according to size criteria were relatively low (46%), in most of the patients (60.7%), the lesion size stabilized after SBRT. A stable lesion is considered beneficial for a patient whose lesion was progressive before the treatment and would have progressed if the treatment had not been implemented. The response rates, including SD, were not statistically different, 61% according to RECIST criteria and 64% according to combined criteria.

In this study, the median survival duration was not reached. The 1- and 2-year overall survival rates were 96% and 67%, respectively. In order to achieve at least 2 years of follow up, we included some patients treated at the beginning of our SBRT practice at which time the patients were treated at a lower dose than in our current practice. However, if we were to report on the complete cohort treated at our center [6], which included 99 lesions in 72 patients until April 2010, the median progression-free survival was 10.5 months, reached by patients who had failed multiple regimens of chemotherapy. The overall survival rate was 72% at 1 year and 65% at 2 years. These survival results are consistent with those previously reported for patients with unresectable liver metastases [5,7,8,18]. These encouraging results should be considered with the selection criteria for the patients eligible for SBRT in mind. These patients usually have good performance status with long disease history, metastases only in the liver or a limited number of other metastases.

**Figure 7:**
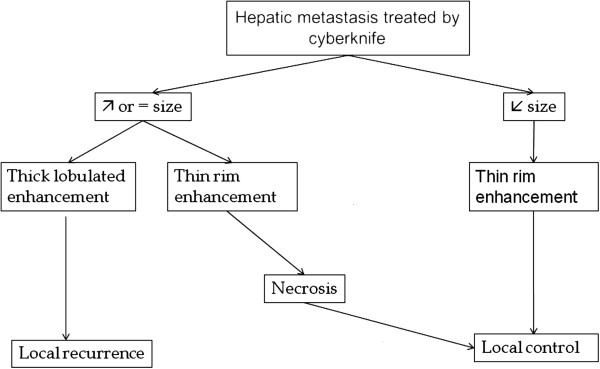
Algorithm using combined criteria for assessing local response for liver metastasis treated by SBRT.

A strategy for the assessment of local response of hepatic metastasis to SBRT is proposed in the algorithm below (Figure 7). In ambiguous cases, follow-up studies would either show the resolution of treatment-related inflammatory changes or an area of persistent enhancement or a residual continuously growing tumor. 

This retrospective study has the usual limitations associated with this type of studies. Histopathological confirmation of imaging findings after therapy was not attempted because of the potential for sampling error. Furthermore, a biopsy was considered too invasive without significant effect on future management. The patient sample was limited to the patients who had at least 2 years of follow-up and lesions larger than 1 cm. Response was assessed on the data from successive follow-ups conducted during at least 2 years. SD based on imaging at 2 years was considered local control without histopathological confirmation.

The response assessment criteria were developed based on retrospective observation performed by one radiologist. Because the described lobular enhancement pattern is subject to interpretation, it is necessary to further confirm their validity prospectively in a larger population of patients and with many radiologists.

## Conclusions

SBRT is an emerging technique for treatment of unresectable liver malignancies, especially liver metastases. The interpretation of post therapeutic imaging features may be challenging for radiologists. The size-based RECIST criteria are not always appropriate in interpreting imaging follow-up of local treatments such as SBRT. Instead we developed a new set of criteria based on a characteristic enhancement pattern on contrast CT combined with the size-based RECIST. These new combined criteria, which need further studies to be validated, may prevent some of the errors that may result from misinterpretation with size-based criteria alone.

## Competing interests

The authors declare that they have no competing interests.

## Authors’ contributions

HJ, XM, ST, LC conceived the study. HJ collected data. HJ drafted the manuscript. XM, AA, SD, FB, ST, AK, LC and EL participated in coordination and helped to draft the manuscript. AK and ET performed the statistical analyses. EL, AA and LC provided mentorship and edited the manuscript. All authors have read and approved the final manuscript.

## References

[B1] Jenkinson MD Haylock B Shenoy A Husband D Javadpour M Management of cerebral metastasis: evidence-based approach for surgery, stereotactic radiosurgery and radiotherapy Eur J Cancer 2011 47 649 655 10.1016/j.ejca.2010.11.033 21196112

[B2] Feuvret L Noel G Nauraye C Garcia P J-Mazeron J [Conformal index and radiotherapy] Cancer Radiother 2004 8 108 119 10.1016/j.canrad.2003.12.002 15132144

[B3] Ruers T Bleichrodt RP Treatment of liver metastases, an update on the possibilities and results Eur J Cancer 2002 38 1023 1033 10.1016/S0959-8049(02)00059-X 11978527

[B4] Sawrie SM Fiveash JB Caudell JJ Stereotactic body radiation therapy for liver metastases and primary hepatocellular carcinoma: normal tissue tolerances and toxicity Cancer Control 2010 17 111 119 20404794 10.1177/107327481001700206

[B5] Fuss M Thomas CR Jr Stereotactic body radiation therapy: an ablative treatment option for primary and secondary liver tumors Ann Surg Oncol 2004 11 130 138 10.1245/ASO.2004.10.907 14761915

[B6] Vautravers-Dewas C Dewas S Bonodeau F Adenis A Lacornerie T Penel N Lartigau E Mirabel X Image-guided robotic stereotactic body radiation therapy for liver metastases: is there a dose response relationship? Int J Radiat Oncol Biol Phys 2011 81 e39 47 10.1016/j.ijrobp.2010.12.047 21377292

[B7] Ambrosino G Polistina F Costantin G Francescon P Guglielmi R Zanco P Casamassima F Febbraro A Gerunda G Lumachi F Image-guided robotic stereotactic radiosurgery for unresectable liver metastases: preliminary results Anticancer Res 2009 29 3381 3384 19661360

[B8] Goodman KA Wiegner EA Maturen KE Zhang Z Mo Q Yang G Gibbs IC Fisher GA Koong AC Dose-escalation study of single-fraction stereotactic body radiotherapy for liver malignancies Int J Radiat Oncol Biol Phys 2010 78 486 493 10.1016/j.ijrobp.2009.08.020 20350791

[B9] Goyal K Einstein D Yao M Kunos C Barton F Singh D Siegel C Stulberg J Sanabria J Cyberknife stereotactic body radiation therapy for nonresectable tumors of the liver: preliminary results HPB Surg 2010 2010 pii 309780 10.1155/2010/309780 PMC2905697 20689733

[B10] Miller AB Hoogstraten B Staquet M Winkler A Reporting results of cancer treatment Cancer 1981 47 207 214 10.1002/1097-0142(19810101)47:1<207::AID-CNCR2820470134>3.0.CO;2-6 7459811

[B11] Chung EP Herts BR Linnell G Novick AC Obuchowski N Coll DM Baker ME Analysis of changes in attenuation of proven renal cysts on different scanning phases of triphasic MDCT AJR Am J Roentgenol 2004 182 405 410 10.2214/ajr.182.2.1820405 14736671

[B12] Nishino M Jagannathan JP Ramaiya NH Van den Abbeele AD Revised RECIST guideline version 1.1: What oncologists want to know and what radiologists need to know AJR Am J Roentgenol 2010 195 281 289 10.2214/AJR.09.4110 20651182

[B13] Choi H Charnsangavej C de Castro Faria S Tamm EP Benjamin RS Johnson MM Macapinlac HA Podoloff DA CT evaluation of the response of gastrointestinal stromal tumors after imatinib mesylate treatment: a quantitative analysis correlated with FDG PET findings AJR Am J Roentgenol 2004 183 1619 1628 10.2214/ajr.183.6.01831619 15547201

[B14] Coenegrachts K Magnetic resonance imaging of the liver: New imaging strategies for evaluating focal liver lesions World J Radiol 2009 1 72 85 10.4329/wjr.v1.i1.72 21160723 PMC2999307

[B15] Caretti V Zondervan I Meijer DH Idema S Vos W Hamans B Bugiani M Hulleman E Wesseling P Vandertop WP Noske DP Kaspers G Molthoff CF Wurdinger T Monitoring of tumor growth and post-irradiation recurrence in a diffuse intrinsic pontine glioma mouse model Brain Pathol 2011 21 441 451 10.1111/j.1750-3639.2010.00468.x 21159008 PMC8094295

[B16] Cheebsumon P Velasquez LM Hoekstra CJ Hayes W Kloet RW Hoetjes NJ Smit EF Hoekstra OS Lammertsma AA Boellaard R Measuring response to therapy using FDG PET: semi-quantitative and full kinetic analysis Eur J Nucl Med Mol Imaging 2011 38 832 842 10.1007/s00259-010-1705-9 21210109 PMC3070082

[B17] Padhani AR Koh DM Diffusion MR imaging for monitoring of treatment response Magn Reson Imaging Clin N Am 2011 19 181 209 10.1016/j.mric.2010.10.004 21129641

[B18] Rusthoven KE Kavanagh BD Cardenes H Stieber VW Burri SH Feigenberg SJ Chidel MA Pugh TJ Franklin W Kane M Gaspar LE Schefter TE Multi-institutional phase I/II trial of stereotactic body radiation therapy for liver metastases J Clin Oncol 2009 27 1572 1578 10.1200/JCO.2008.19.6329 19255321

[B19] Lee MT Kim JJ Dinniwell R Ley J Lockwood G Wong R Cummings B Ringash J Tse RV Knox JJ Dawson LA Phase I study of individualized stereotactic body radiotherapy of liver metastases J Clin Oncol 2009 27 1585 1591 10.1200/JCO.2008.20.0600 19255313

[B20] Takayasu K Arii S Matsuo N Yoshikawa M Ryu M Takasaki K Sato M Yamanaka N Shimamura Y Ohto M Comparison of CT findings with resected specimens after chemoembolization with iodized oil for hepatocellular carcinoma AJR Am J Roentgenol 2000 175 699 704 10.2214/ajr.175.3.1750699 10954453

[B21] Dromain C de Baere T Elias D Kuoch V Ducreux M Boige V Petrow P Roche A Sigal R Hepatic tumors treated with percutaneous radio-frequency ablation: CT and MR imaging follow-up Radiology 2002 223 255 262 10.1148/radiol.2231010780 11930075

[B22] Ebied OM Federle MP Carr BI Pealer KM Li W Amesur N Zajko A Evaluation of responses to chemoembolization in patients with unresectable hepatocellular carcinoma Cancer 2003 97 1042 1050 10.1002/cncr.11111 12569604

[B23] Kalb B Chamsuddin A Nazzal L Sharma P Martin DR Chemoembolization follow-up of hepatocellular carcinoma with MR imaging: usefulness of evaluating enhancement features on one-month posttherapy MR imaging for predicting residual disease J Vasc Interv Radiol 2010 21 1396 1404 10.1016/j.jvir.2010.05.015 20688534

[B24] Lencioni R Llovet JM Modified RECIST (mRECIST) assessment for hepatocellular carcinoma Semin Liver Dis 2010 30 52 60 10.1055/s-0030-1247132 20175033 PMC12268942

[B25] Chun YS Vauthey JN Boonsirikamchai P Maru DM Kopetz S Palavecino M Curley SA Abdalla EK Kaur H Charnsangavej C Loyer EM Association of computed tomography morphologic criteria with pathologic response and survival in patients treated with bevacizumab for colorectal liver metastases JAMA 2009 302 2338 2344 10.1001/jama.2009.1755 19952320 PMC4139149

[B26] Siegel CL Fisher AJ Bennett HF Interobserver variability in determining enhancement of renal masses on helical CT AJR Am J Roentgenol 1999 172 1207 1212 10.2214/ajr.172.5.10227490 10227490

[B27] Wong CY Salem R Raman S Gates VL Dworkin HJ Evaluating 90Y-glass microsphere treatment response of unresectable colorectal liver metastases by [18F]FDG PET: a comparison with CT or MRI Eur J Nucl Med Mol Imaging 2002 29 815 820 10.1007/s00259-002-0787-4 12029557

[B28] Olsen CC Welsh J Kavanagh BD Franklin W McCarter M Cardenes HR Gaspar LE Schefter TE Microscopic and macroscopic tumor and parenchymal effects of liver stereotactic body radiotherapy Int J Radiat Oncol Biol Phys 2009 73 1414 1424 10.1016/j.ijrobp.2008.07.032 18990508

[B29] Herfarth KK Hof H Bahner ML Lohr F Höss A van Kaick G Wannenmacher M Debus J Assessment of focal liver reaction by multiphasic CT after stereotactic single-dose radiotherapy of liver tumors Int J Radiat Oncol Biol Phys 2003 57 444 451 10.1016/S0360-3016(03)00586-8 12957256

[B30] Yamasaki SA Marn CS Francis IR Robertson JM Lawrence TS High-dose localized radiation therapy for treatment of hepatic malignant tumors: CT findings and their relation to radiation hepatitis AJR Am J Roentgenol 1995 165 79 84 10.2214/ajr.165.1.7785638 7785638

